# Medical and Physical Works in the Library of the Lane Theological Seminary

**Published:** 1837

**Authors:** 


### Art. VIII.—Medical and Physical Works in the Library of Lane Theological Seminary.

The learned Professor Stowe of this institution, situated on the *Walnut Hills*, adjoining Cincinnati, lately made a voyage to France, Germany and England, to purchase books. On visiting the library, a short time since, we learned that it contains 8,000 volumes, written or printed in the following languages:—Hebrew, Arabic, Ethiopic, Syriac, Chaldee, Persian, Hindoo, Hieroglyphic Egyptian, Greek, Latin, Italian, Spanish, French, German, Romaic, Dutch and English. They have cost the Seminary $8,000; but many of them were presented, and they are estimated to be worth more than twice that sum. The professor, being a ripe and liberal scholar, did not limit himself strictly to theology; and among the works, there are several of interest to the medical profession, some of which, as being rare in the West, we shall enumerate; that such of our friends as are fond of extended literary research, may know where to find them.

The Transactions of the Royal Society of London, from its foundation in 1665 to 1800; abridged by Hutton, Shaw & Pearson, in eighteen large quarto volumes.

This work contains a great number of valuable papers on medicine and its associate sciences.

Lectures on Comparative Anatomy, from preparations in the Hunterian Collection, by Sir Everard Home; in six volumes, quarto, with splendid engravings.

This work was compiled by Home, from the MSS. of his brother-in-law, John Hunter; which MSS., as mentioned in a former number of our journal, he afterwards destroyed, that he might the more successfully appropriate the enviable and hard-earned fame of his great master, to himself!

Cuvier's Animal Kingdom, translated by several distinguished Naturalists; with numerous additions and many plates, in sixteen 8vo volumes.

Buffon's Works, complete, with Anatomical Descriptions, by Daubenton, in 44 volumes, octavo, with numerous plates. French.

The last edition of Sir Charles Bell on the Nervous System; illustrated with engravings far superior to those in the American editions of his works, quarto.

Lizars' Anatomical Plates, colored, folio.

Maud's Botanic Garden, embracing all the Plants cultivated in Great Britain; quarto; with colored engravings; many volumes, and still in the course of publication.

Sir William Jardine's Naturalist's Library; in 12mo.; extending through many volumes, with beautifully colored plates.

A Collection of Colored Engravings, with letter press, containing the Botany of J. J. Rousseau,; twenty-six volumes, octavo. In French.

The Encyclopædia Britannica, and the Encyclopedia Metropolitana, more than sixty volumes, quarto, both in the course of publication.

Sketches of Bedlam, being an account of the intellectual phenomena of the most remarkable maniacs, who have at different times been confined to the London Insane Hospitals.

An Essay on the Curative Powers of Animal Magnetism, by Professor C. A. F. Kluge, of Berlin. In German.

When in Hamburg, in September last, Professor Stowe saw the learned and indefatigable Dr. Julius, whose visit to the United States, was mentioned, two years ago in our journal,

Dr. J. confirmed the report, that his extensive collection of American books and pamphlets was consumed, in New York, by the *great* fire. As a traveller of such enlightened zeal does not often visit the United States, we cannot but deeply regret the destruction of his large accumulations of our literature and science. D.

